# CHALLENGES FOR BLIND AND VISION IMPAIRED USERS OF A VISUAL QUESTION ANSWERING TOOL: IMPLICATIONS FOR AGING ADULTS

**DOI:** 10.1093/geroni/igz038.3306

**Published:** 2019-11-08

**Authors:** Nathan Davis, Bo Xie, Danna Gurari

**Affiliations:** The University of Texas at Austin, Austin, Texas, United States

## Abstract

Recent technological developments provide individuals with vision impairment the transformative ability to upload pictures they take and promptly receive descriptions from remote workers. This study aimed to: identify challenges for visually impaired individuals to use such technology to obtain health-related information and provide recommendations for crowd-workers and the future development of assistive artificial intelligence (AI) design. In spring and summer of 2019, we analyzed 265 images of medication packages submitted by users of a visual question answering (VQA) application called VizWiz -- a smartphone application that provides near realtime assistance to visually impaired users by employing crowd-workers. We developed a 4-category coding scheme to analyze image quality, with two independent coders achieving excellent intercoder reliability (85%-95%). Of the 265 images, we found less than half were legible (46%), contained clear indicators for information sought (40%), or had minimum background noise (40%); while only a small percentage contained complete information (6%). Through thematic analysis of the data, we also highlight seven challenges with queries submitted by vision impaired users . Based on our findings, we make recommendations for the future design of VQA technologies, such as VizWiz, for visually impaired users. We also suggest that there is both great need and potential for user-centered design research to significantly enhance such assistive technologies. While this study did not focus exclusively on data submitted by aging adults, many VizWiz users are, in fact, aging adults, and such assistive technologies have strong implications for the design of assistive technology for this age group.

